# Percutaneous Vertebroplasty and Facet Blocking for Treating Back Pain Caused by Osteoporotic Vertebral Compression Fracture

**DOI:** 10.1155/2020/5825317

**Published:** 2020-08-11

**Authors:** Yongquan Cheng, Xiaoliang Wu, Jiawei Shi, Hui Jiang

**Affiliations:** Division of Spine Surgery, Department of Orthopedics, Nanfang Hospital, Southern Medical University, Guangzhou, China

## Abstract

**Methods:**

Clinical and radiological data of 204 patients were reviewed. The patients were divided into Group A (PVP alone) and Group B (PVP and FB combined therapy) according to treatments. Back pain was evaluated with Visual Analog Scale (VAS) and Oswestry Disability Index (ODI). The operation, fluoroscopic exposure time, and bone cement leakage were recorded. The *χ*^2^ test, Student's *t*-test, and repeated measures analysis of variance were used to compare the differences between the two groups.

**Results:**

There were 125 patients in Group A and 79 patients in Group B. Their baseline characteristics were similar (*P* > 0.05). The mean VAS scores of Group A and Group B were 7.03 and 7.21 at admission, 4.7 and 3.2 at 1 day after operation, 4.0 and 3.0 at 3 months, and 2.2 and 2.2 at 12 months after operation, respectively. The mean ODI scores of Group A and Group B were 30.9 and 29.8 at admission, 17.6 and 17.7 at 3 months, and 10.5 and 10.9 at 12 months after operation, respectively. The mean operation time and fluoroscopic exposure time of Group A (35.6 minutes and 7.2 seconds, respectively) was significantly shorter than that of Group B (45.7 minutes and 11.7 seconds, respectively, *P* < 0.01). The incidence of bone cement leakage and new fractures after operation did not have statistically significant difference between groups.

**Conclusion:**

PVP and FB combined therapy could provide better pain relief than PVP alone in short term after operation in patients with OVCFs associated back pains.

## 1. Introduction

Osteoporotic vertebral compression fractures (OVCFs) are a major complication of osteoporosis and are becoming more prevalent as population aging. Back pain associated with OVCFs will limit the mobility of patients and cause several problems such as deep vein thrombosis, decubitus ulcer, and hypostatic pneumonia [1].

There are several verified treatments for OVCFs, including conservative treatment, open surgery, and percutaneous vertebroplasty (PVP). PVP was first introduced for treating vertebral hemangioma in 1987 [2]. Shortly thereafter it has been adopted by many authors for treating symptomatic OVCFs as a minimal invasive surgery [3–5]. It could provide rapid pain relief and improvement of life quality. Although PVP is effective in most patients, someone still has back pain after PVP or its effectiveness had been doubted [6]. It is postulated that pains associated with OVCFs may arise not just from vertebral body but also from posterior elements [7]. Therefore, facet blocking (FB) and medial branch blocking would be beneficial for alleviating back pain associated with OVCFs [8, 9]. A prospective study showed that PVP produced better pain relief than FB in the short term, but the difference in pain relief between these two techniques was insignificant in the long term (1 month to 12 months) [10]. Kim et al. investigated PVP and FB combined therapy and found that it was a profitable method for OVCFs [3]. But no one has compared the efficacy of PVP and FB combined therapy with PVP alone in English literature.

Thus, this study is conducted to compare the clinical and radiologic outcomes of these two therapies.

## 2. Methods

This retrospective study had been approved by the Ethics Committee of Nanfang Hospital and written form of consents had been provided by all participants. All patients who were diagnosed as OVCFs with back pain and admitted for percutaneous vertebroplasty from January 1, 2017, to December 31, 2018, were enrolled. XR and MRI were performed to confirm newly onset of OVCFs. Exclusion criteria included neurologic deficit, coagulation dysfunction, spinal infection, and loss to follow-up.

Among the 225 enrolled patients, 204 were included in this study while 21 were excluded due to loss to follow-up. The medical records including charts and radiological findings were collected. Patients were divided into two groups according to treatments: PVP alone (Group A) and PVP and FB combined therapy (Group B). Clinical data including age, sex, bone mineral density (BMD) measured by dual energy absorptiometry (DEXA), Visual Analog Scale (VAS), and Oswestry Disability Index (ODI) scores except sex item were collected. For VAS rating, the subject is asked to place a mark somewhere on a 10 cm line to assess present pain. The two extremes are labeled to correspond to the absolute minimum (0 cm) and the absolute maximum pain (10 cm) that could ever be experienced [11]. ODI is a 10-item questionnaire scoring 0 to 5 to assess patient's home and work life and analgesic requirements. Then it is calculated as percentage, with a high score indicating high level of disability [12]. In this study, one item (sex activity) was omitted because the patients were old and sexually inactive. The time points of VAS were at admission, 1 day, and 3 and 12 months after treatment, while the time points of ODI were at admission and 3 and 12 months after treatment. Radiographs at admission, 1 day, and 3 and 12 months after treatment were collected.

The PVP was performed through bilateral transpedicular approach in the prone position. After localization of the fractured vertebral body and local anesthesia with 1% lidocaine (v/v), an 11-gauge needle was inserted into the pedicle under the guidance of anterior-posterior and lateral fluoroscopic views. After the needle tip was placed into the anterior one-third or one-fourth of the fracture vertebral body, the inner needle was taken out and 3–5 ml of high-viscosity polymethyl methacrylate (PMMA) bone cement was injected under continuous fluoroscopic guidance until the bone cement was close to the cortical margin or spinal canal.

The FB was performed just after PVP in Group B. A 23-gauge needle was used for FB bilaterally. Under guidance of fluoroscope, the needle was inserted into the facet capsules of the adjacent vertebral bodies above and below the fractured one. The mixture solution was composed of 20 ml of 2% lidocaine, 20 ml of normal saline, and 2 ml of betamethasone. Then 2 ml of mixture solution was injected into each capsule. The operation time, fluoroscopic exposure time, blood loss during operation, and leakage of bone cement were recorded for both groups.

Calcium carbonate 600 mg and calcitriol 0.25 *μ*g were administered daily to patients of both groups after operation. Two hours after operation, patients were mobilized to walk around bed without brace. Cox-2 inhibitors such as Celecoxib or Parecoxib would be given as required if patients had surgical site pain within 3 days after operation. Back muscle exercise was taught by nurses before discharge.

Statistical analysis was performed with SPSS software (version 23.0). Quantitative results were presented as mean ± standard deviation. The *χ*^2^ test, Student's *t*-test, and repeated measures analysis of variance were used to compare the differences between the two groups. A multivariate logistic regression model with a backward stepwise method was used to evaluate the risk factors of new fractures after treatment. *P* < 0.05 was considered as statistically significant.

## 3. Results

Group A had 125 patients, while Group B had 79 patients. The demographic characteristics of patients are shown in Table 1. The male-to-female patient ratio was 37 : 167. The mean age of patients was 71.8 ± 9.1 years (range, 50–98 years; median 71 years). The mean T-score of BMD was 3.0 ± 0.46. The VAS and ODI score at admission were 7.10 ± 1.12 and 30.51 ± 7.18, respectively. These baseline features of two groups had no statistically significant differences.

The mean operation time of Group A (35.6 ± 5.9 minutes) was significantly shorter than that of Group B (45.7 ± 5.9 minutes, *P* < 0.01). Similarly, the mean fluoroscopic exposure time of Group A (7.2 ± 3.2 seconds) was significantly shorter than that of Group B (11.7 ± 6.3 seconds, *P* < 0.01). There was no significant difference in blood loss during operation between the two groups (4.8 ± 2.2 ml vs. 5.3 ± 3.1 ml, *P*=0.33). Student's *t*-test was used for comparing these parameters.

**Figure 1:**
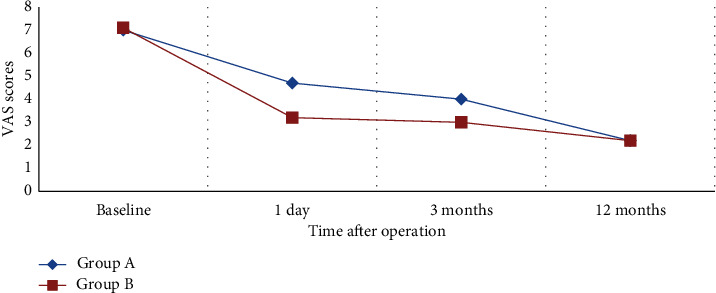
Comparison of Visual Analog Scale scores in two groups after operation.

The mean VAS scores of Group A and Group B were 4.7 ± 1.0 and 3.2 ± 0.8 at 1 day, 4.0 ± 0.8 and 3.0 ± 0.7 at 3 months, and 2.2 ± 0.6 and 2.2 ± 0.7 at 12 months after operation, respectively (Figure 1). For both groups, the VAS scores after operation significantly decreased when compared with baseline data (repeated measures analysis of variance, *P* < 0.01). The VAS scores showed greater improvement in Group B at 1 day and 3 months after operation compared with Group A, but there was no statistically significant difference at 12 months after operation (Student's *t*-test, Figure 1).

The mean ODI scores of Group A and Group B were 17.6 ± 4.6 and 17.7 ± 5.5 at 3 months and 10.5 ± 2.6 and 10.9 ± 3.2 at 12 months after operation, respectively (Table 2). The improvement of ODI scores between groups did not differ significantly (repeated measures analysis of variance).

The leakage of bone cement occurred in 10 and 8 patients of Group A and Group B, respectively. The incidence of this complication had no statistically significant difference between groups (*χ*^2^ test, *P*=0.764). All of these patients were asymptomatic. None of them needed further treatment. For new fractures confirmed by XR after operation during follow-up, 16 occurred in Group A and 12 occurred in Group B. The incidence of new fractures was not statistically significant different between groups (*χ*^2^ test, *P*=0.655). A multivariate logistic regression model with a backward stepwise methods showed that low BMD at admission was the only risk factor for new fractures after treatment even after adjusting confounding factors (*P* < 0.05).

## 4. Discussion

Osteoporosis is becoming more and more prevalent as population aging. In elderly over 70 years old, the prevalence of osteoporosis is about 40% in China [13]. OVCFs are one of the most common and severe complications resulting from osteoporosis. It was estimated that there were 700,000 OVCFs every year in the United States [14]. Back pain associated with OVCFs would cause loss of mobility, depression, and pulmonary dysfunction [4]. The concurrent treatment strategies of OVCFs include conservative therapy, open surgery, and minimal invasive cement augmentation surgeries, namely, PVP or balloon kyphoplasty [10].

Cement augmentation can provide immediate, significant, and sustained pain relief in OVCFs patients. It can also rapidly improve physical function and quality of life [15, 16]. Therefore, PVP surgeries have been performed extensively. But the very benefit of cement augmentation itself was doubted by two studies published in 2009. They compared vertebroplasty with a sham procedure. Surprisingly, both improvement in pain and disability from osteoporotic compression fractures were similar in patients treated with vertebroplasty and those treated with simulated vertebroplasty [6, 17]. These studies raised ardent debates about the effectiveness of vertebroplasty. Another concern is that some OVCFs patients still have back pain after vertebroplasty [9].

One possible explanation for these questions is that back pain of OVCFs may have multiple generators. The pain can be derived from acute fracture and inflammation proximal to the fracture site. Vertebroplasty can reduce the micromovement in the fracture site as well as neurolysis within the vertebral body due to heat generated by PMMA [18]. But in some patients with OVCFs, the back pain may also arise from posterior elements rather than fracture alone. The facet joints may be abnormally stressed due to overflexion after thoracic compression fracture, which may serve as a secondary pain generator [15]. A biomechanics model confirmed that the posterior elements of the vertebral column must subluxate cephalad or caudad in response to deformity of a vertebral body [7]. A radiologic study has demonstrated associated facet signal change on MRI in acute/subacute vertebral compression fractures, further supporting this theoretical model [19].

Thus medial branch blocking and FB were introduced to treat OVCFs related back pain. Kim et al. found that physical examination after FB was the most reliable method to confirm the most painful level among multiple OVCFs sites, and PVP and FB combined treatment had the advantages of low risk and short duration of procedure with a high chance to result in pain relief and early mobilization [3]. But they did not compare the combined treatment with PVP alone in terms of efficacy and efficiency. In a retrospective study, 53 patients with axial back pain from OVCFs were treated with medial branch block. The medial branch block provided significant pain relief and functional recovery to the patients with OVCFs complaining of continuous facet joint pain after vertebroplasty or conservation treatment [9]. A third of patients technically suitable for vertebroplasty responded beneficially to facet joint injection [8]. A prospective randomized controlled trial compared PVP with FB for severe pain due to OVCFs in 206 patients. The results showed that PVP produced better pain relief than facet blocking in the short term, but the difference in pain relief between these two techniques was insignificant in the long term [10]. Those results showed the extensive existence of soft tissue injury in OVCF patients, and the relative advantage of PVP and FB combined therapy. But it is unknown whether PVP and FB combined therapy could provide more benefit than PVP alone in OVCFs patients with back pain. This single center retrospective study was performed to elucidate this matter.

The mean VAS scores of Group A (PVP alone) and Group B (PVP and FB combined therapy) were 7.03 and 7.21 at admission, 4.7 and 3.2 at 1 day after operation, 4.0 and 3.0 at 3 months, and 2.2 and 2.2 at 12 months, respectively. The improvement of VAS scores for 1 day and 3 months after operation in Group B was statistically greater than that in Group A. This result confirmed that PVP and FB combined therapy can provide better pain relief in OVCFs patients in short term. Further studies focusing on quality of life after PVP and FB combined therapy would reveal more benefit of it, like study conducted by Imai et al. [20]. A retrospective study published in Chinese also found similar short-term benefits of PVP and FB combined therapy, but the sample size was smaller and there was lack of long-term follow-up [21]. Our study found that, after 1 year of operation, there was no statistically significant difference in VAS and ODI scores between groups. This could be attributed to the stabilization of spinal column and short-term effectiveness of local anesthetic agents and steroids [22–25]. The mean ODI scores of Group A and Group B were 30.9 and 29.8 at admission, 17.6 and 17.7 at 3 months, and 10.5 and 10.9 at 12 months after operation, respectively. These results also suggested the similarity of long-term pain relief between groups.

In our study, the mean operation time of Group A (35.6 minutes) was significantly shorter than that of Group B (45.7 minutes). The mean fluoroscopic exposure time of Group A (7.2 seconds) was also shorter than Group B (11.7 seconds). This was reasonable because FB took some time in addition to PVP. In the prospective study performed by Wang et al., the mean operation time of FB group and PVP group was 22.5 and 35.3 minutes, respectively [10]. FB would slightly increase the operation time and fluoroscopic exposure to patients and surgeons. This should be informed to OVCFs patients before operation and weighed against better pain relief in short term after operation.

The most common complication of PVP is cement leakage, which includes leakage into surrounding tissue, paravertebral vein embolism, intradiscal leakage, and leakage into spinal canal. The cumulative incidence could be as high as 40%, although majority of them do not produce any clinical symptoms [26]. In this study, the incidence of this complication did not differ significantly in statistics between groups, and none of these patients were symptomatic. Intravertebral cleft, cortical disruption, low cement viscosity, and high volume of injected cement may be the risk factors of cement leakage [27].

New fractures after PVP would occur in more than 10% of patients and could be symptomatic requiring further treatment [28]. The incidence in this study was about 14%, which was close to literature reports, and the incidences in the two groups were similar. Low BMD at admission was found to be the risk factor for new fractures in this study. A meta-analysis also demonstrated that low bone mineral density, the presence of multiple treated vertebrae, and a history of steroid usage were associated with the new OVCFs after vertebroplasty [29]. These risk factors should be considered in further analysis of our data. PVP would increase the incidence of new vertebral fractures. This might be explained by a shift in mechanical load of the spine after the bone cement was injected, increasing stress in adjacent vertebral bodies [28, 30].

There are a few limitations in this study. First, as a retrospective study, there might be several biases that affect treatment effects between groups. Further prospective controlled trial comparing PVP and FB combined therapy with PVP alone is required. Second, control group treated with FB only was lacking in this study. Third, PVP and FB slightly increased the operation time and medical cost, which should be informed to patients. Fourth, the follow-up was only 12 months, which would not be long enough to detect new fractures. Nonetheless, this study is the first large size one to investigate the benefit of PVP and FB combined therapy compared with PVP alone for managing back pain of OVCFs patients.

## 5. Conclusion

In patients with back pain due to OVCFs, PVP and FB combined therapy could provide better pain relief than PVP alone in short term after operation. Although FB would slightly increase operation time and fluoroscopic exposure, it is still worth to perform together with PVP if back pains generating from posterior elements are suspected.

## Figures and Tables

**Table 1:** Baseline demographic and clinical data of the two groups.

Characteristics	Group A (*n* = 125)	Group B (*n* = 79)	*P* value
Age, years	70.8 ± 8.9	72.9 ± 9.3	0.966
Sex			0.458
Male	25	12	
Female	100	67	
BMD	3.04 ± 0.45	2.94 ± 0.47	0.143
VAS score	7.0 ± 1.1	7.2 ± 1.1	0.256
ODI score	30.9 ± 7.0	29.8 ± 7.3	0.285

BMD, bone mineral density; VAS, Visual Analog Scale; ODI, Oswestry Disability Index. Age, BMD, VAS score, and ODI score were analyzed with Student's *t*-test, while sex was analyzed with *χ*^2^ test.

**Table 2:** Comparison of Oswestry Disability Index scores in two groups after operation.

Time after operation	Group A (*n* = 125)	Group B (*n* = 79)	*P* value
3 months	17.6 ± 4.6	17.7 ± 5.5	0.863
12 months	10.5 ± 2.6	10.9 ± 3.2	0.321

## Data Availability

The data used to support this study can be made available from the corresponding author upon request.
